# Innovative Trinuclear Copper(I)-Based Metal–Organic Framework: Synthesis, Characterization, and Application in Laser-Induced Graphene Supercapacitors

**DOI:** 10.3390/nano16030155

**Published:** 2026-01-23

**Authors:** Hiba Toumia, Yu Kyoung Ryu, Habiba Zrida, Alicia De Andrés, María Belén Gómez-Mancebo, Natalia Brea Núñez, Fernando Borlaf, Ayoub Haj Said, Javier Martinez

**Affiliations:** 1 Laboratory of Interfaces and Advanced Materials (LIMA), Faculty of Sciences of Monastir, University of Monastir, Monastir 5019, Tunisia; hiba.toumia@alumnos.upm.es (H.T.); ayoub.hajsaid@crmn.rnrt.tn (A.H.S.); 2 Instituto de Sistemas Optoelectrónicos y Microtecnología, Universidad Politécnica de Madrid, Av. Complutense 30, 28040 Madrid, Spain; y.ryu@upm.es; 3 Departamento de Física Aplicada e Ingeniería de Materiales, Escuela Técnica Superior de Ingenieros Industriales, Universidad Politécnica de Madrid, C/José Gutiérrez Abascal 2, 28006 Madrid, Spain; 4 Instituto de Ciencia de Materiales de Madrid, Consejo Superior de Investigaciones Científicas, C/Sor Juana Inés de La Cruz 3, Cantoblanco, 28049 Madrid, Spain; ada@icmm.csic.es; 5 División de Química, Departamento de Tecnología, Centro de Investigaciones Energéticas, Medioambientales y Tecnológicas, Av. Complutense 40, 28040 Madrid, Spain; mariabelen.gomez@ciemat.es; 6 Unidad de Hidrogeociencias Ambientales, Departamento de Medio Ambiente, Centro de Investigaciones Energéticas, Medioambientales y Tecnológicas, Av. Complutense 40, 28040 Madrid, Spain; 7 Unidad de Espectroscopía, Departamento de Tecnología, Centro de Investigaciones Energéticas, Medioambientales y Tecnológicas, Av. Complutense 40, 28040 Madrid, Spain; 8 Microelectronics and Nanotechnology Research Center, Technopole de Sousse, Sousse 4054, Tunisia; 9 Departamento de Ciencia de Materiales, Escuela Técnica Superior de Ingenieros Caminos, Canales y Puertos, Universidad Politécnica de Madrid, C/Profesor Aranguren s/n, 28040 Madrid, Spain

**Keywords:** metal–organic framework, H_2_NDI-H, copper (I), laser-induced graphene, supercapacitors

## Abstract

Optimizing efficient electrode materials that combine high energy density, rapid charge transport, and excellent cycling stability remains a challenge for advanced supercapacitors. Here, we report the synthesis of an innovative copper(I)-based metal–organic framework (MOF), Cu_3_(NDI)_3_, prepared via a simple solvothermal method using N,N’-bis(3,5-dimethylpyrazol-4-yl)-naphthalene diimide (H_2_NDI-H) as a linker. Structural analyses (XRD, FTIR, SEM, EDX, and BET) confirmed the formation of a highly crystalline, porous MOF. Integration of this MOF into laser-induced graphene (LIG) matrices yielded hybrid electrodes with enhanced structural characteristics and electrochemical activity, compared to its only-LIG counterpart. Electrochemical studies (CV, CD, EIS) revealed that the LIG–MOF electrode exhibited the highest performance, delivering a specific capacitance of 4.6 mF cm^−2^ at 0.05 mA cm^−2^, and an areal energy density of 60.03 μWh cm^−2^ at a power density of 1292.17 μW cm^−2^, outperforming both LIG and MOF–LIG configurations. This enhancement arises from the synergetic interaction between the conductive LIG network and the redox-active Cu_3_(NDI)_3_ framework, highlighting the potential of LIG–MOF hybrids as next-generation materials for high-performance supercapacitors.

## 1. Introduction

The global energy crisis and environmental urgency demand sustainable and efficient energy storage solutions [1]. Supercapacitors offer both higher energy storage capability and faster charge/discharge rates than conventional capacitors, bridging the gap between capacitors and batteries [2,3]. Recent miniaturized versions show hundred- to thousand-fold energy density improvements in the same volume [4]. Supercapacitors outperform lithium-ion batteries in charge/discharge speed, power density, lifespan, and flexibility [5]. Their flexible designs use conductive carbon networks as combined current collectors/electrodes, creating lightweight structures ideal for wearable electronics [6,7]. Finally, novel electrolytes that pair the flexibility requirements of the electrodes and expand their working voltage window such as cellulose-based gels and ionogels are being actively explored [8,9].

Laser-induced graphene (LIG) has emerged as an attractive electrode material for flexible electric double-layer capacitors (EDLCs), combining high surface area, excellent conductivity, and mechanical flexibility [10,11]. Produced through direct photothermal or/and photochemical conversion of diverse polymer precursors [12,13,14,15,16] into 3D porous graphene networks under ambient conditions [17,18], this synthesis method enables direct 2D patterning using commercial laser systems while offering tunable porosity through laser parameter modulation. The transformation of the precursor as the carbon source into graphene-like materials and the patterning of structures with the device geometry are performed simultaneously in a single step.

However, the electric double-layer capacitance (EDLC) mechanism of pristine LIG results in limited intrinsic capacitance [19,20,21]. To address this challenge, recent studies have demonstrated two primary modification strategies: (i) heteroatom doping (e.g., N, S, or P) to enhance pseudocapacitance contribution through surface redox reactions [22,23,24], and (ii) hybridization with electroactive nanomaterials such as transition metal oxides [25,26], metal phosphates [27], metal–organic frameworks (MOFs) [28,29], or MXenes [30,31]. These composite systems synergistically combine the electric double-layer capacitance (EDLC) of LIG with Faradaic charge storage mechanisms, leading to significantly improved energy storage performance. Inside the group of electroactive nanomaterials described above regarding strategy (ii), metal–organic frameworks are crystalline porous materials composed of metal-cluster secondary building units (SBUs) bridged by organic linkers [32], exhibiting exceptional properties including ultrahigh surface areas (≤10.400 m^2^/g), tunable porosity (0.5–10 nm), and remarkable thermal and chemical stability [33]. These hybrid materials can be synthesized through various methods ranging from conventional solvo-/hydrothermal processes to advanced microwave, electrochemical, and mechanochemical approaches [34,35], enabling diverse applications in energy storage [36,37], gas storage/separation [38], catalysis [39], sensing [40,41], and biomedical fields [42].

Trinuclear copper–pyrazolate (Cu_3_Pz_3_) complexes are particularly attractive for MOF synthesis as they combine predictable coordination geometry, strong Cu–Cu interactions, and high structural robustness [43,44,45,46,47]. These features enable the design of porous frameworks with tunable pore size distributions and excellent chemical stability, both of which are crucial for efficient ion storage and rapid electrolyte penetration in supercapacitor electrodes. In addition to providing a stable scaffold, the trinuclear arrangement increases the density of electroactive sites, thereby enhancing charge accumulation at the electrode–electrolyte interface. When coupled with naphthalene diimide (NDI)-based ligands, the electrochemical functionality of the framework is further improved. Owing to their extended π-conjugation, strong electron-accepting ability, and intrinsic redox activity [48,49], NDI units participate in Faradaic processes and facilitate rapid electron transfer through the conductive network. By further integrating these MOFs with laser-induced graphene, the resulting hybrid materials combine the high conductivity and mechanical robustness of graphene with the hierarchical porosity and electroactive functionality of MOFs. This synergy not only enhances overall capacity by providing both double-layer and pseudocapacitive contributions but also ensures fast ion and electron transport, ultimately leading to superior electrochemical performance compared to pristine LIG and positioning this composite electrode as a promising candidate for next-generation supercapacitors.

In the present study, we demonstrate the successful synthesis of Cu_3_(NDI)_3_, a novel trinuclear copper(I)-based metal–organic framework, through a solvothermal method using N,N′-bis(3,5-dimethylpyrazol-4-yl)-naphthalene diimide as a structure-directing ligand. Then, we used the MOF to dope LIG using two different approaches: deposition of MOF after formation of LIG (LIG-MOF) and deposition of MOF on the polyimide precursor, with simultaneous irradiation of both elements to produce the doped LIG (MOF-LIG). Finally, LIG-MOF and MOF-LIG materials were used as electrodes to fabricate microsupercapacitors. All the materials—the MOF, LIG, LIG-MOF composite and MOF-LIG composite—were thoroughly characterized by structural, chemical, and electrochemical methods, which confirmed the enhancement of the laser-induced graphene properties after the addition of Cu_3_(NDI)_3_ MOF due to the combination of the MOF abundant redox-active sites and the LIG’s excellent electrical conductivity and hierarchical porosity. As supercapacitor, the LIG-MOF device presented the best performance, achieving the highest specific capacitance and areal energy density of, respectively, 4.6 mF cm^−2^ at 0.05 mA cm^−2^ and 60.03 μWh cm^−2^ at a power density of 1292.17 μW cm^−2^.

## 2. Materials and Methods

### 2.1. Materials

Copper nitrate hemi (pentahydrate (Cu(NO_3_)_2_·2.5H_2_O, 1,4,5,8-Naphthalenetetracarboxylic dianhydride), N,N-dimethylformamide (DMF), methanol (MeOH), ethanol (EtOH), and water (H_2_O). H_2_NDI is synthesized similarly to published procedures [50]. All chemicals and reagents used in this study were purchased from Sigma-Aldrich (Darmstadt, Germany) with a purity of 98% or higher and were used as received without further purification.

A 125 µm thick polyimide (PI) film (DuPont^TM^ Kapton^®^ HN, Lake Villa, IL, USA) was used as a precursor of LIG and a 60 µm thick adhesive polyimide (PI) film (Tesa^®^, Norderstedt, Germany) was used for the assembly of the devices. Sulfuric acid (H_2_SO_4_, 98%), adhesive copper tape, and silver paint were purchased from RS PRO. 

### 2.2. Structural Characterization

X-ray diffraction (XRD) studies were conducted using a Cu Kα radiation source (λ = 1.54 Å) on an X’Pert Pro diffractometer (Malvern-Panalytical, Malvern, Worcestershire, UK) operating at 45 kV and 40 mA. XRD data were collected in the θ-θ configuration within the angular range of 2.5° to 80°, with a step size of 0.050°. X-ray fluorescence (XRF) measurements were performed with an Axios Wavelength Dispersive X-ray Fluorescence spectrometer with a Rh tube (Malvern-Panalytical, Malvern, Worcestershire, UK). The morphology of the samples was examined using scanning electron microscopy (SEM) on an FEI Inspect^TM^ F50 (FEI Company, Hillsboro, OR, USA) at an accelerating voltage of 5 kV. Energy-dispersive X-ray analysis (EDX) was performed at an accelerating voltage of 30 kV to conduct elemental analysis. The measurements were carried out using an FEI APREO S scanning electron microscope equipped with an energy-dispersive X-ray (EDX) detector (FEI Company, Hillsboro, OR, USA). Fourier Transform Infrared (FTIR) spectroscopy was performed using a PerkinElmer Spectrum Two FT-IR spectrometer (PerkinElmer, Shelton, CT, USA) with attenuated total reflectance to analyze absorption bands in the region of 4000 to 400 cm^−1^. Thermogravimetric analysis (TGA) data were collected using a STA 6000 (PerkinElmer, Shelton, CT, USA) from 30 °C to 800 °C at a heating rate of 10 °C min^−1^ under flowing N_2_ and air atmospheres. The specific surface area (SSA) and porosity of the samples were determined using the Brunauer–Emmett–Teller (BET) and Barrett–Joyner–Halenda (BJH) method with an ASAP 2020 (Micromeritics, Norcross, GA, USA). Additionally, the distribution of pore sizes from the N_2_ adsorption–desorption isotherms was evaluated by Barrett–Joyner–Halenda (BJH) method. Raman spectra were acquired using a 532 nm solid state laser, a Horiba (iHR-320) monochromator (Horiba, Kyoto, Japan) with a 600 L/mm grating, the detector Peltier cooled CCD (Synapse, Horiba, Kyoto, Japan), and an Olympus microscope with a x20 objective, an optical density of 0.5, 1 s of acquisition time, and 30 accumulations.

### 2.3. Electrochemical Characterization

An Autolab PGSTAT204 potentiostat/galvanostat system (Metrohm AG, Herisau, Switzerland) and the Nova 1.11 software were employed to conduct cyclic voltammetry (CV) and galvanostatic charge–discharge (GCD) measurements on the fabricated devices. For electrochemical impedance spectroscopy (EIS) measurements, a FRA32M frequency response analyzer module of the PGSTAT204 system was used.

Electrochemical performance was evaluated in a two-electrode configuration on symmetric in-plane micro supercapacitors (MSCs) using a 1M H_2_SO_4_ aqueous electrolyte at room temperature and under ambient conditions.

EIS data were recorded with a sinusoidal signal at an amplitude of 10 mV and a frequency range of 0.01–100 KHz.

The device areal capacitance (*C_A_*, in mF cm^−2^) was extracted from the GCD measurements using the following formula:

(1) $$C_{A}\;=\;\frac{I_{discharge}}{S\times (\frac{dv}{dt})}$$

where *I_discharge_* is the constant discharge current, *S* is the total area of both the positive and negative electrodes, and *dV/dt* is the slope of the discharge curve.

The areal energy density (*E_A_*, in μWh cm^−2^) is calculated using the following formula:

(2) $$E_{A}=\frac{1}{2}\times C_{A}\times \frac{V^{2}}{3600}$$

where *V* = *V*_drop_ is the voltage of the next point in the discharge curves.

The areal power density (*P_A_*, in μW cm^−2^) is calculated using the following formula:

(3) $$P_{A}=\frac{E_{A}}{∆t}\times 3600$$

where Δ*t* is the discharge time (in seconds).

### 2.4. Preparation of Trinuclear Copper Cu-MOF with Naphtalene: Cu_3_(NDI)_3_

In this work, H_2_NDI was synthesized following a previously reported procedure [50] with minor adjustments, and the key steps are now explicitly described to improve reproducibility. Briefly, 1,4,5,8-naphthalenetetracarboxylic dianhydride (NTCDA) was used as the starting material and converted into the corresponding diimide ligand (H_2_NDI) via a controlled condensation reaction in high-boiling DMF. Specifically, 1,4,5,8-naphthalenetetracarboxylic dianhydride (1 mmol) and 4-amino-3,5-dimethylpyrazole (2 mmol) were placed in a 50 mL round-bottom flask, followed by the addition of 20 mL of anhydrous DMF. The reaction mixture was stirred under reflux at 150 °C for 12 h under a N_2_ atmosphere. After completion of the reaction, the resulting precipitate was collected by filtration, washed successively with DMF and diethyl ether (Et_2_O), and dried under vacuum to afford the product as a yellow powder (yield: 85%). The obtained H_2_NDI ligand was then directly used for MOF synthesis without further modification.

**Figure 1:**
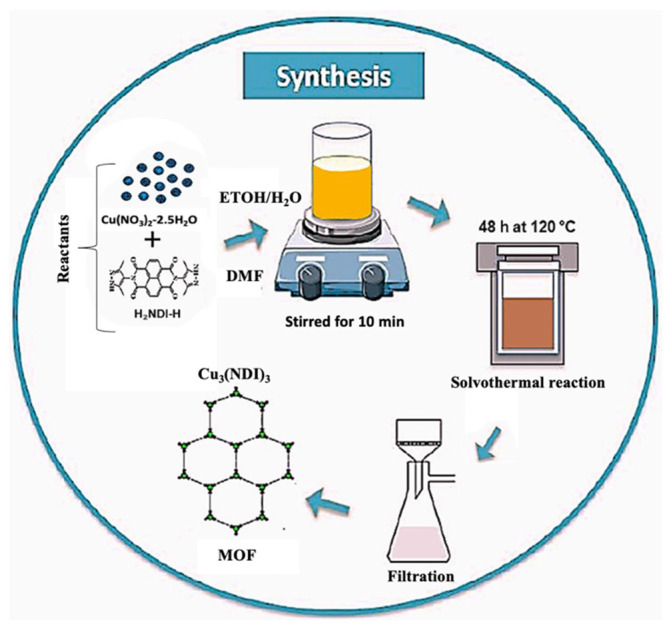
Scheme with the followed experimental method for the solvothermal synthesis of Cu_3_(NDI)_3_ MOF.

Subsequently, a light brown MOF powder, Cu_3_(NDI)_3_ (tris(bis(3,5-dimethylpyrazol-4-yl)-naphthalene diimide)-tri-copper) was synthesized by reacting Cu(NO_3_)_2_·2.5H_2_O (0.150 g, 0.5 mmol) with N,N’-bis(3,5-dimethylpyrazol-4-yl)-naphthalene diimide (H_2_NDI-H) (0.114 g, 0.25 mmol) ligand in a solvent mixture of DMF:ethanol:water with 10:3:2 ratios at 120 °C for 48 h [51]. The product was isolated, washed with DMF and methanol, and dried under vacuum. The structure Cu_3_(C_24_H_16_N_6_O_4_)_3_ was confirmed by powder X-ray diffraction, as shown later in the corresponding characterization section. A scheme summarizing the synthesis process of the MOF is shown in Figure 1.

### 2.5. Synthesis of the Electrodes

**Figure 2:**
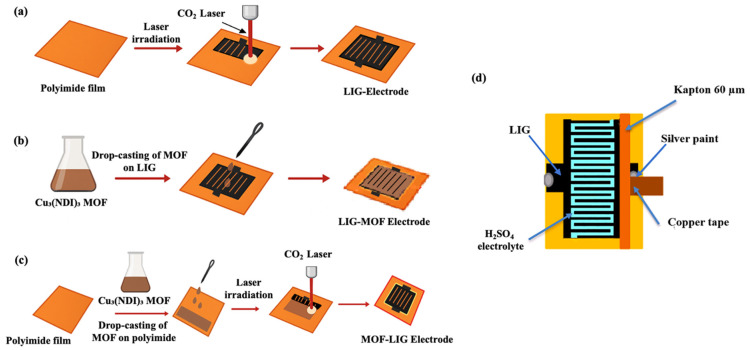
Schemes illustrating the fabrication processes of the (**a**) LIG, (**b**) LIG-MOF, (**c**) and MOF-LIG electrodes. (**d**) Scheme with the assembly of the microsupercapacitor components to produce the final device. This assembly process was the same for the three types of electrodes shown in (**a**–**c**).

A commercial 10.6 μm CO_2_ laser was used to produce LIG electrodes on polyimide (PI) precursor via photothermal process (Figure 2a). The electrode patterns were designed using Inkscape (vector graphics software). The laser had a focused beam diameter of 100 µm [52], with adjustable power up to 40 W and scan speed up to 600 mm/s. The interdigitated electrode design consisted of 20 fingers, each 10 mm long × 0.6 mm wide, with 450 µm spacing. For this study, the laser operated at 80 mm/s of scan speed and 6% of maximum power, 2.4 W, with the distance between the laser and the polyimide film being maintained at a focal length of 8 mm, under ambient conditions. These lasing parameters were chosen based on the optimization process of LIG fabrication from Kapton film developed in previous works of our group [52,53]. After laser scribing and pyrolysis, shape controllable LIG samples were successfully obtained. Through the text, this type of electrode, only-LIG, will be denominated as LIG. The scheme of the fabrication route is shown in Figure 2a.

The synthesis of LIG-Cu_3_(NDI)_3_ electrodes consisted of the following approach. After fabrication (Figure 2a), the LIG electrode was functionalized by drop-casting an ethanol dispersion of Cu_3_(NDI)_3_ onto its surface. The resulting LIG-Cu_3_(NDI)_3_ composite electrode was then dried for 1 h. Through the text, this type of electrode will be denominated as LIG-MOF to denote the prior formation of the LIG and the subsequent doping with MOF. The scheme of the fabrication route is summarized in Figure 2b.

The synthesis of Cu_3_(NDI)_3_-LIG electrodes consisted of the following approach. First, a homogeneous ethanol dispersion of Cu_3_(NDI)_3_ was drop-casted onto a pristine polyimide substrate, forming a uniform MOF layer. After drying for 1 h, laser irradiation converted the PI precursor coated with Cu_3_(NDI)_3_ into the Cu_3_(NDI)_3_-LIG composite electrode. Through the text, this type of electrode will be denominated as MOF-LIG, to denote the prior deposition of MOF on the polyimide precursor and subsequent simultaneous irradiation of both materials to form the final doped LIG composite. The scheme of the fabrication route is summarized in Figure 2c.

### 2.6. Microsupercapacitor Assembly Process

For the electrolyte, 2.0 mL of 98% H_2_SO_4_ was diluted in 20 mL ultrapure water, stirred for 60 min at room temperature until obtaining a homogeneous solution.

Once fabricated, the electrodes were contacted with the current collectors at the side LIG squares: adhesive copper tape, with silver paint between the copper and the LIG to enhance electrical conductivity. Then, the current collectors were insulated with 60 µm adhesive Kapton to prevent their contact with the electrolyte. Finally, 0.15 mL of electrolyte was applied on the device active area. This assembly process to produce the full supercapacitor, with its scheme represented in Figure 2d, was followed in the same manner for the three types of electrodes: LIG, LIG-MOF, and MOF-LIG.

The bare LIG supercapacitors were fabricated as a control to compare with the other two types of electrodes-based devices and check the improvement in the performance of the LIG-MOF and MOF-LIG supercapacitors thanks to the MOF doping effect. The LIG-MOF and MOF-LIG supercapacitors were fabricated to study the difference in the performance of the devices when the MOF is deposited after the fabrication of the LIG and when the MOF is deposited on the polyimide precursor and irradiated while the LIG is being formed.

## 3. Results and Discussion

### 3.1. Structural Characterization of Cu_3_(NDI)_3_ MOF

Cu_3_(NDI)_3_ was synthesized following the experimental procedures outlined in the corresponding section above. The resulting material underwent comprehensive characterization to evaluate its structural properties, microstructural features, and phase purity. Additionally, critical synthesis parameters were systematically optimized to ensure the production of nano-sized MOF particles with the desired characteristics.

**Figure 3:**
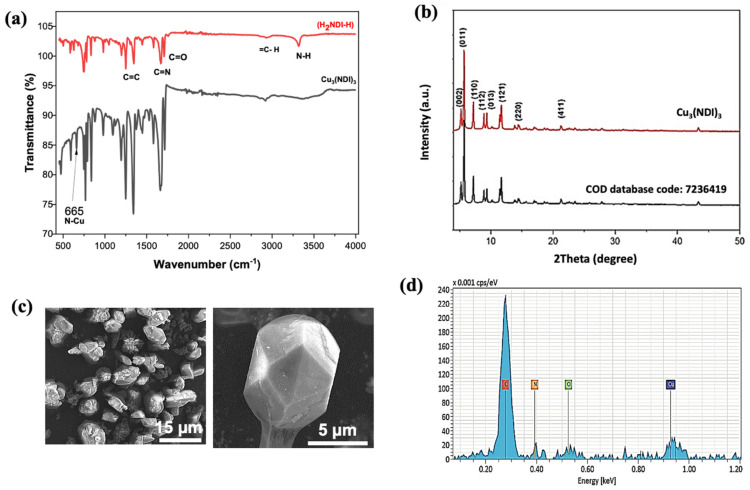
(**a**) Comparative FTIR spectra of Ligand H_2_NDI-H (red curve) and MOF Cu_3_(NDI)_3_ (black curve). (**b**) XRD patterns of Cu_3_(NDI)_3_ (red curve) and reference (black curve). (**c**) SEM images of Cu_3_(NDI)_3_ at 5000× (**left**) and 20.000× (**right**) magnification. (**d**) EDX spectrum of Cu_3_(NDI)_3_ MOF.

The FTIR spectrum of Cu_3_(NDI)_3_ (Figure 3a, black curve) exhibits characteristic bands at 2978–2900 cm^−1^ (C-H stretching), 1703 cm^−1^ (C=O), 1673 cm^−1^ (C=N), and 1447–1196 cm^−1^ (C=C), consistent with the H_2_NDI-H ligand (Figure 3a, red curve). The disappearance of the N-H stretching band at 3324 cm^−1^ and the emergence of a new Cu-N vibration at 655 cm^−1^ [54] confirm ligand coordination through nitrogen atoms, demonstrating successful MOF formation (Figure 3a, black curve). This spectroscopic result verifies the deprotonation of the ligand and subsequent metal–ligand bond formation.

Powder X-ray diffraction analysis was conducted to investigate the crystalline structure of the Cu_3_(NDI)_3_ metal–organic framework, as illustrated in Figure 3b. The experimental XRD patterns (red curve) were obtained using a diffractometer with a Cu Kα radiation source and compared to simulated patterns derived from the crystallographic data of a reference single crystal (CIF COD 7236419) (black curve). A strong agreement was observed between the experimental and simulated patterns [55], with closely matching characteristic peaks at 2θ angles of 21.25°, 14.29°, 11.47°, 9.4°, 8.9°, 7.17°, 5.68°, and 5.22°.

Rietveld refinement, carried out using the FullProf software (FullProf Suite, Windows 64 bits, August 2025), revealed that the MOF crystallizes in the I-4 space group. The lattice parameters were determined to be a = 17.3321 Å, b = 17.3321 Å, c = 33.8106 Å, and α = β = γ = 90°, confirming a highly ordered three-dimensional framework. The calculated unit cell volume suggests the presence of potential porosity within the structure, considering the spatial arrangement of the framework components. A summary with the crystallographic data for Cu_3_(NDI)_3_ MOF is presented in Table S1 (Supplementary Materials File).

Scanning electron microscopy images reveal that Cu_3_(NDI)_3_ crystallizes in well-defined octahedral morphologies with truncated facets (Figure 3c). A group of MOF crystals is shown in Figure 3c (left), and an individual crystal in Figure 3c (right). Quantitative analysis indicates an average crystal surface area of 38.64 ± 0.05 μm^2^.

Complementary EDX spectrum (Figure 3d) confirms the expected stoichiometric composition, with characteristic emission lines for C Kα (0.27 keV), N Kα (0.40 keV), O Kα (0.52 keV), and Cu Kα (0.95 keV). The material exhibits exceptional purity (>99.9 at%), with elemental ratios matching theoretical values within ±0.3 at%.

The porous characteristics of the synthesized Cu_3_(NDI)_3_ material were evaluated through low-temperature (77 K) nitrogen physisorption measurements. As illustrated in Figure S1a (Supplementary Materials File) the adsorption–desorption isotherm exhibits a characteristic Type IV profile according to IUPAC classification, confirming the mesoporous nature of the framework. Quantitative analysis revealed a Brunauer–Emmett–Teller (BET) specific surface area of 238.35 m^2^ g^−1^, with the total pore volume of pores 0.126 cm^3^ g^−1^. The pore size distribution, calculated using the Barrett–Joyner–Halenda (BJH) method, indicated a narrow mesopore distribution centered at 2.11 nm (Figure S1b, Supplementary Materials File).

### 3.2. Structural Characterization of Electrodes

**Figure 4:**
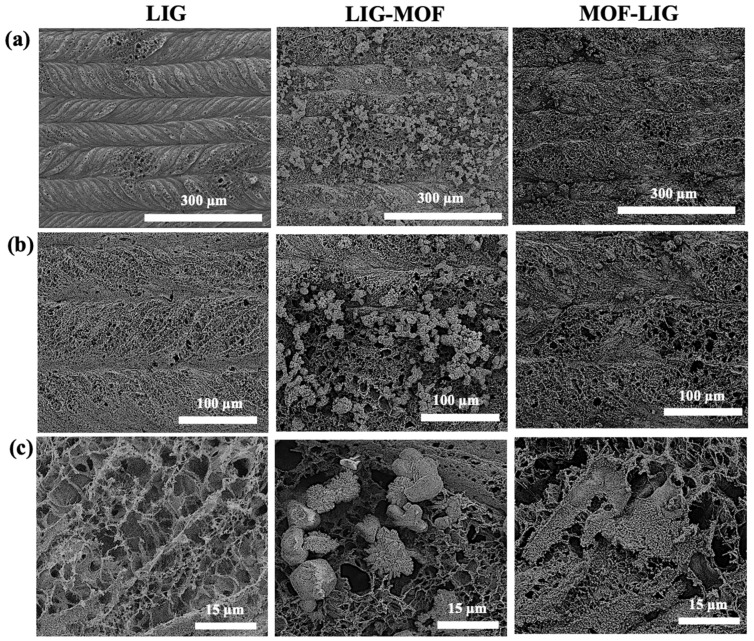
SEM images of LIG (**left column**), LIG-MOF (**center column**), and MOF-LIG (**right column**) electrodes. For (**a**–**c**), the top, center, and bottom row images correspond to magnifications of 500×, 1000× and 5000×, respectively.

Figure 4 displays SEM images of the three different types of electrodes at various magnifications. In the LIG sample, the trajectory of the laser movement is clearly visible as the polymer substrate undergoes expansion and bending during laser irradiation (Figure 4a,b, left images). This deformation produces distinctive C-shaped lines when the laser moves from left to right, while the opposite pattern emerges when the laser moves in the reverse direction. The LIG films exhibit a porous structure, marked by thin graphene sheets oriented perpendicular to the polyimide (PI) substrate. The morphology of LIG reveals a hierarchical architecture composed of interconnected, multilayered flakes. From a higher magnification image, shown in Figure 4c (left), these flakes feature small, short fibers at their edges. Similar morphological characteristics have been documented in previous studies on laser-induced graphene produced from polyimide using CO_2_ lasers [56,57].

The SEM images of the LIG-MOF electrode are displayed in the center column of Figure 4. From the image of the lowest magnification, shown in Figure 4a (center), it can be observed that the trajectory of the laser movement on the LIG surface is still visible in the regions where it is not covered by the MOF material. Cu_3_(NDI)_3_ grains exhibiting an octahedral morphology are uniformly dispersed across the LIG sheet network. This hierarchical structure is anticipated to facilitate efficient electron transport and enhance energy storage performance. Within the LIG-MOF electrode, the three-dimensional porous network of LIG acts as a structural support, ensuring the even distribution of Cu_3_(NDI)_3_ crystals throughout the interconnected graphene framework via interfacial interactions (Figure 4b,c, center images). Furthermore, the high electrical conductivity of LIG positions it as an inherently effective material for electric double-layer capacitor (EDLC) energy storage. This property also aids in optimizing electrolyte diffusion to the active surfaces of Cu_3_(NDI)_3_, thereby improving overall electrochemical performance.

The right column of Figure 4 depicts significant morphological changes in the MOF-LIG electrode following laser treatment, which corresponds when the MOF and the polyimide precursor were irradiated simultaneously. In the SEM images of the lower magnifications (Figure 4a,b, right images), an expanded and exfoliated framework panorama is observed. This transformation mainly arises from carbonization, expansion due to the rapid release of gases during laser irradiation, as well as exfoliation of the MOF structure. As a result, a three-dimensional porous network is formed, expected to facilitate efficient electron transport and to improve energy storage performance. From the highest magnification SEM image of Figure 4c (right), it can be checked that, under this fabrication approach, a highly porous network and a high amount of surface area are achieved as well. In addition, the interconnected graphene network provides structural stability and ensures uniform dispersion of the exfoliated MOF components.

Figure S2a–c (Supplementary Materials File) show the N_2_ adsorption–desorption isotherms and Figure S2d–f (Supplementary Materials File), the pore size distribution plots of LIG, LIG-MOF, and MOF-LIG electrodes, respectively. From the N_2_ adsorption–desorption isotherms, it is verified that all samples exhibit type IV isotherms, indicative of mesoporous structures. The BET specific surface area and porous characteristics of the three types of electrodes, together with the ones from only MOF sample (Figure S1a,b in Supplementary Materials), calculated using the Barrett–Joyner–Halenda (BJH) method, are summarized in Table 1.

Regarding the BET surface area analysis, among LIG, LIG-MOF, and MOF-LIG electrodes, the LIG-MOF composite delivers the highest specific surface area of 232.678 m^2^ g^−1^ and the highest pore volume of 0.165 cm^3^ g^−1^. From Table 1, it can be seen that LIG-MOF composite has similar SSA than only-MOF material and similar pore volume than only-LIG material, which means that, thanks to the synergy of both materials, the composite electrode gains the best characteristics of each. This result will be correlated later in Section 3.3, where it is demonstrated that the supercapacitor with the best performance is the one fabricated using the LIG-MOF electrodes. This also ratifies the beneficial effect of doping laser-induced graphene with Cu_3_(NDI)_3_ metal–organic framework to enhance its functionality. The enlarged surface area of LIG-MOF compared to bare LIG provides more active sites and better ion accessibility, which is advantageous for electrochemical energy storage. The MOF-LIG composite exhibits the lowest surface area of 138.659 m^2^ g^−1^, likely due to partial structural collapse of MOF during CO_2_ laser pyrolysis, as it was also observed from the SEM images in Figure 4c. However, it is later proven in Section 3.3 that the performance of supercapacitors fabricated with MOF-LIG electrodes is still better than the devices that employ LIG electrodes, indicating that the increase in the active sites thanks to the presence of MOF (positive result) overcomes the decrease in SSA (negative result).

**Figure 5:**
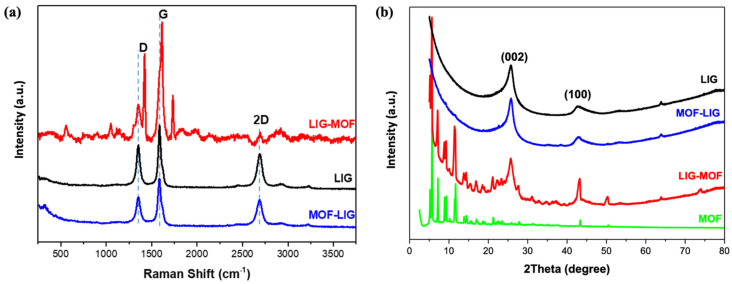
(**a**) Raman spectra of LIG (black curve), LIG-MOF (red curve), and MOF-LIG (blue curve). The vertical dashed blue lines are added as a visual reference to locate the D, G, and 2D peak characteristics of graphene. (**b**) XRD patterns of LIG (black curve), MOF (green curve), LIG-MOF (red curve), and MOF-LIG (blue curve). In (**a**,**b**), in each case, all spectra are represented at the same scale, but just displaced along the vertical direction to make their visualization and comparison to the readers easier.

Raman spectroscopy measurements were performed to confirm the successful synthesis of laser-induced graphene and the subsequent functionalization via Cu_3_(NDI)_3_ MOF to obtain composite electrodes (Figure 5a). The spectra for both LIG and MOF-LIG exhibit three characteristic peaks of graphene-like materials. The D band at ~1350 cm^−1^ is associated with structural defects or disorder in the carbon lattice, while the G band at ~1580 cm^−1^ arises from the in-plane stretching vibrations of sp^2^-hybridized carbon atoms. The presence of a distinct 2D band at ~2650 cm^−1^ is a hallmark of few-layer graphene, with its sharpness inversely related to the number of layers [58]. In the case of the LIG electrode spectrum (Figure 5a, black curve), the clear and sharp 2D peak confirms the successful formation of a graphenic structure via laser-induced transformation of the PI substrate. Furthermore, the structural quality is quantified by the intensity ratio of the D to G bands (I_D_/I_G_), which is 0.55 for LIG, indicating good crystallinity [58]. The intensity ratio of the 2D to G bands (I_2D_/I_G_) is less than 1, an expected value for the multilayer nature of the LIG material [59]. Finally, the presence of the D’ band (around ~1620 cm^−1^) is detected, which is associated with defects due to intravalley scattering near the Dirac point [60]. Regarding the Raman spectrum of the MOF-LIG composite (Figure 5a, blue curve), MOF-related bands are not detected. Here, we remember the fact that, for the case of MOF-LIG electrodes, the MOF is deposited on the polyimide precursor and both are then irradiated at the same time to produce and dope the LIG simultaneously. The used laser writing parameters have induced changes in the MOF crystal structure such as partial breaking of the linkers or amorphization, which can hinder the Raman signal of the MOF-related peaks [61]. However, from the SEM images (Figure 4, right column), the incorporation of the MOF on the LIG was checked. Second, demonstrated later in Section 3.3, the enhancement in the performance of the supercapacitor based on MOF-LIG electrodes, compared to the device made with bare LIG electrodes, indicates that the doping of LIG with MOF has been effective and beneficial. Finally, regarding the LIG-related peaks analysis, the MOF-LIG spectrum shows that the D, G, and 2D bands are very similar to the ones displayed in the LIG spectrum. The D’ band is also present, and the I_D_/I_G_ ratio of 0.45 is similar as well, corroborating that, despite the MOF incorporation during the irradiation process, a good quality LIG is formed.

Raman spectroscopic analysis of the LIG-MOF composite (Figure 5a, red curve) confirms the presence of characteristic MOF bands, verifying its successful incorporation in the LIG and the preservation of its crystallinity since, in this case, the MOF was deposited on the LIG after its fabrication. Specific bands attributable to the NDI ligand are identified, including the C=C stretching vibrations of aromatic rings (1051 and 1607 cm^−1^), the C=O vibrations of the imide at 1733 cm^−1^, the aromatic C–H stretching modes at 2895 cm^−1^, and the C=N stretching vibration of the pyrazole ring observed at 1424 cm^−1^. Furthermore, the band at 564 cm^−1^ is assigned to Cu–N coordination, confirming the presence of the metal–organic framework [62]. In this case, the D and G peaks corresponding to LIG appear as shoulders of the 1424 cm^−1^ and 1607 cm^−1^ MOF peaks, and the D’ band is not visible. A striking feature is the very weak intensity of the 2D peak. Its intensity shrinkage is a consequence of the MOF doping effect and indicates a strong interaction between LIG and MOF [63].

X-ray diffraction (XRD) analysis was performed on LIG, pure MOF, MOF-LIG, and LIG-MOF samples to examine their crystalline structure (Figure 5b). The obtained XRD patterns are displayed in Figure 5b. The spectra of LIG, LIG-MOF, and MOF-LIG exhibit distinctive peaks at around 26° and 43°, corresponding to the (002) and (100) crystallographic planes of hexagonal graphite, respectively [64,65].

The XRD patterns of pure MOF (green curve) sample and the LIG-MOF (red curve) composite show multiple sharp diffraction peaks characteristic of MOF crystals, indexed to the (002), (011), (110), (112), (113), (121), (220), and (411) planes. These reflections align well with the simulated XRD pattern of single-crystal MOF reported in the literature, confirming the preservation of the MOF crystalline framework in the composite materials [66]. Finally, the XRD pattern of the MOF-LIG (blue curve) composite is marked by the absence of MOF crystal-related peaks, for the same reason that was explained from the corresponding Raman spectrum (Figure 5a, blue curve).

XRF analysis showed that the composition in the bare LIG electrodes is mainly carbon, at 90.2 wt%, with no detectable Cu. In LIG-MOF electrodes, the carbon content decreases to 80 wt% while Cu content rises to 6.5 wt%, confirming successful MOF deposition. For MOF-LIG electrodes, laser pyrolysis increases the carbon content to 89.2 wt% and lowers Cu content to 1.36 wt%, indicating partial MOF decomposition and incorporation of Cu species into the LIG carbon matrix.

### 3.3. Electrochemical Performance of Supercapacitors

The electrochemical performance of LIG, LIG-MOF, and MOF-LIG MSCs was assessed using cyclic voltammetry (CV), galvanostatic charge–discharge (GCD), and electrochemical impedance spectroscopy (EIS) in a two-electrode configuration to measure symmetric devices.

To achieve device fabrication processes with good reproducibility for each of the type of electrodes studied in this work—LIG, LIG-MOF, and MOF-LIG—5 to 7 samples were fabricated to optimize the assembling of the supercapacitor components, until we obtained three consecutive devices that showed similar performance, a result that reassured us we had reached this optimization. In the Supplementary Material File, Figure S3, the cyclic voltammetry curves, all measured at 500 mV s^−1^, of these three devices with reproducible behavior from the three types of electrodes are provided.

**Figure 6:**
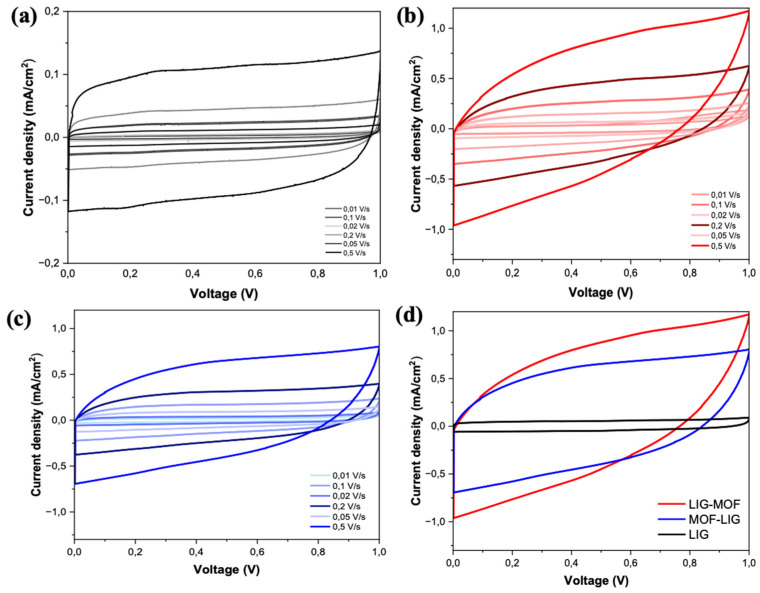
CV curves at varying scan rates of (**a**) LIG MSC, (**b**) LIG-MOF MSC, and (**c**) MOF-LIG MSC. (**d**) CV profiles of LIG (black curve), LIG-MOF (red curve), and MOF-LIG (blue curve) MSCs recorded at a scan rate of 500 mV s^−1^ and put together in the same graph at the same scale to facilitate the direct comparison of their performances.

Figure 6a–c illustrate the CV curves for LIG, LIG-MOF, and MOF-LIG MSCs at scan rates varying from 10 to 500 mV/s. The CV curves of the three types of supercapacitors display a highly symmetrical and rectangular shape, consistent with the behavior of an electric double-layer capacitor (EDLC), as expected in the case of graphene-based materials (Figure 6a), and this shape was kept when MOF material is added to LIG in this work. The retention of this symmetrical shape even at a high scan rate of 500 mV/s demonstrates the electrodes’ exceptional rate capability. The significant improvement in the LIG-MOF and MOF-LIG MSCs’ performance when compared to the LIG device (Figure 6d) is linked to the inclusion of the pseudocapacitive mechanism in the energy storage process due to the incorporation of the Cu_3_(NDI)_3_ MOF. The addition of MOF indicates redox reactions involving the copper ions and the electrochemically active naphthalene diimide (NDI) linker, which undergo reversible oxidation and reduction processes at the electrode-electrolyte interface. Comparable behavior has been documented in studies involving composite materials like CuO-decorated reduced graphene oxide (rGO), LIG/MoS_2_, and MnO_2_/rGO [67,68,69].

**Figure 7:**
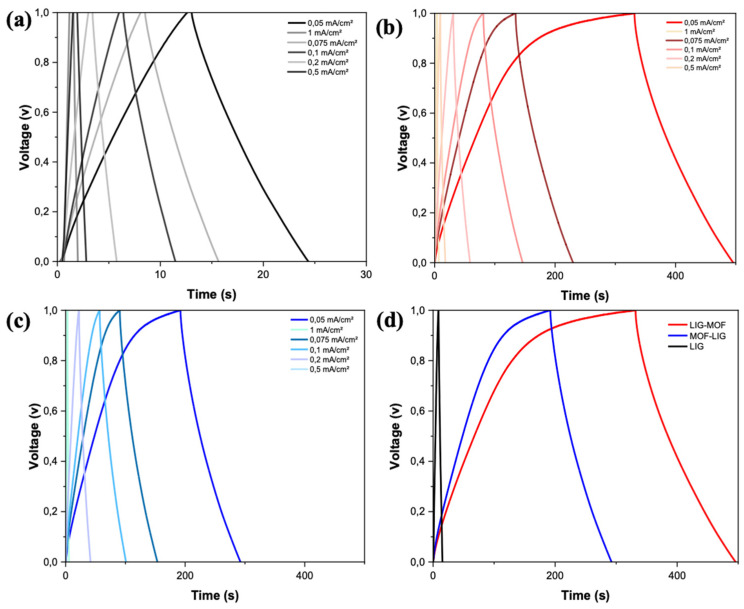
Galvanostatic charge–discharge curves at various currents densities of (**a**) LIG MSC, (**b**) LIG-MOF MSC, and (**c**) MOF-LIG MSC. (**d**) GCD curves for LIG (black curve), LIG-MOF (red curve), and MOF-LIG (blue curve) at a current density of 0.05 mA cm^−2^ and put together in the same graph at the same scale to facilitate the direct comparison of their performances.

The galvanostatic charge–discharge curves of the LIG, LIG-MOF, and MOF-LIG MSCs, measured at current densities ranging from 0.05 to 1 mA cm^−2^, are presented in Figure 7a–c, which show a series of symmetric, triangular-shaped GCD curves characteristic of the electric double-layer capacitor behavior of LIG material (Figure 7a), and this shape was kept in the cases of LIG-MOF and MOF-LIG MSCs (Figure 7b and Figure 7c, respectively). Once more, the significant improvement in the LIG-MOF and MOF-LIG MSCs when compared to the LIG device (Figure 7d) is attributed to the introduction of pseudocapacitance due to the presence of Cu_3_(NDI)_3_.

**Figure 8:**
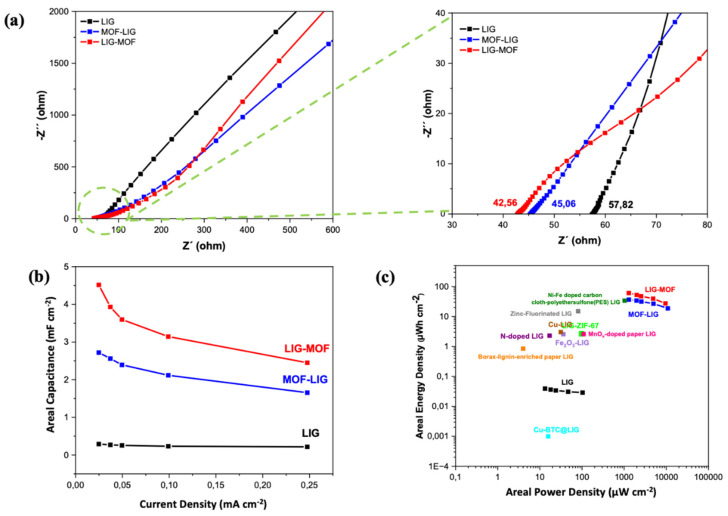
(**a**) Nyquist plots of LIG, LIG-MOF, and MOF-LIG MSCs from EIS measurements. (**b**) Areal capacitance as a function of current density for the LIG, LIG-MOF, and MOF-LIG MSCs. (**c**) Ragone plot of the fabricated supercapacitors in this work and their comparison with previously reported MOF-doped LIG supercapacitors: LIG-ZIF-67 [28], Cu-BTC@LIG [29], borax-lignin-enriched paper LIG [81], N-doped LIG [82], Fe_2_O_3_ LIG [83], MnO_x_-doped paper LIG [84], Cu-LIG [85], zinc-fluorinated LIG [86], and Ni-Fe-doped carbon cloth-polyethersulfone (PES) LIG [87].

To further evaluate the electrochemical performance of the LIG, LIG-MOF, and MOF-LIG MSCs, electrochemical impedance spectroscopy (EIS) was performed. The spectra of the measurements are represented in Figure 8a, and the scheme of the used equivalent electrical circuit is shown in Figure S4 (Supplementary Material File), which consists of an electric series resistance (R_s_), a charge transfer resistance (R_ct_), a Warburg impedance (R_w_), and the capacitance element (C) [70,71]. As illustrated in the augmented inset of the Nyquist plot from Figure 8a (right), the intercepts on the *X*-axis in the high-frequency region, which represents the electrode resistance, are 42.56 Ω for the LIG-MOF MSC and 45.06 Ω for the MOF-LIG MSC, which are lower values than 57. 82 Ω for the LIG MSC. Continuing in the high-frequency region (Figure 8a, right), in the case of the LIG-MOF device spectrum (red curve), an onset of a semicircle behavior is noticed, indicating a faster charge transport between the electrode and electrolyte compared to the LIG and MOF-LIG MSCs. At very high frequencies, the limited time available prevents ions from penetrating the pores, restricting access to only the external surface and enhancing the impact of resistive components [29]. Finally, the low-frequency region represents the Warburg diffusion resistance of the devices. The slope of the Nyquist plot in the low-frequency region, in the case of the LIG-MOF MSC (red curve in Figure 8a, left), exceeds 1, demonstrating rapid electrolyte ion diffusion. To summarize, the EIS results also show the best quality of LIG-MOF device with respect to the LIG and MOF-LIG ones.

The areal capacitance (C_A_) of LIG, LIG-MOF, and MOF-LIG MSCs at different current densities were calculated from the GCD curves using Equation (1), represented in Figure 8b. At the same current density, the LIG-MOF MSC (red curve) exhibits a higher areal capacitance compared to both the LIG MSC (black curve) and the MOF-LIG MSC (blue curve). For instance, at a current density of 0.05 mA cm^−2^, the LIG-MOF MSC achieves a significantly higher C_A_ of 4.6 mF cm^−2^, which is substantially greater than that of the LIG MSC (0.29 mF cm^−2^) and almost 2 times higher than that of the MOF-LIG MSC (2.61 mF cm^−2^). Again, both the LIG-MOF and MOF-LIG MSCs present better performance than the LIG device, pointing out the beneficial effect of doping the LIG with MOF, independently of the fabrication approach.

As the current density increases, the specific capacitance of all three electrodes gradually decreases. This decrease is mainly caused by the limited kinetics of redox reactions within the MOF structure at elevated current densities [29]. When the current density is raised from 0.05 to 1 mA cm^−2^, the capacitance retention of the electrodes remains above 50%, with values of 75.86% for the pure LIG MSC, 55.47% for the LIG-MOF MSC, and 59.26% for the MOF-LIG MSC, demonstrating their robust rate capability. On the other hand, in the presence of MOF within the electrodes, the areal capacitance decreases by approximately 40% of its initial value. This reduction is associated with the degradation of the CuO crystal structure during repeated ion insertion and extraction processes, which diminishes the pseudocapacitive contribution over increasing cycles [72,73].

As a summary of the electrochemical characterization results, Cu_3_(NDI)_3_ MOF incorporates a redox-active naphthalene diimide (NDI) organic linker capable of reversible multi-electron redox reactions, which participates in the charge storage mechanism as well. This contrasts with most reported laser-induced graphene doped with MOF supercapacitors, where capacitance is dominated overall by electric double-layer effects or metal-centered redox activity. Consequently, the superior performances of the LIG-MOF and MOF-LIG MSCs studied in this work reflect a hybrid energy storage contribution, where EDLC mechanism is provided by laser-induced graphene and a pseudocapacitive mechanism is displayed by the MOF, from both metallic ions and organic linker redox activity, which has been rarely explored in previous LIG-based MOF supercapacitor electrodes.

In addition, the following correlation between the material structure and the electrochemical characterization helps to understand why the LIG-MOF and MOF-LIG MSCs present better performance than the LIG MSC and why the LIG-MOF MSC is the best device achieved in this work. The SEM analysis confirmed the effective incorporation of MOF on LIG, both after the drop-casting of MOF on the produced LIG in the case of LIG-MOF, as well as under co-lasing of the polyimide precursor and MOF to form simultaneously LIG doped with MOF in the case of MOF-LIG. The effective doping of LIG in the LIG-MOF and MOF-LIG electrodes was translated to their better electrochemical performance as supercapacitors compared to the only-LIG device. The Raman spectroscopy and XRD analyses from LIG-MOF and MOF-LIG composites showed the presence of the D, G, and 2D peaks from the Raman spectra and the XRD peaks around 26° and 43° ((002) and (100) planes of graphite), indicating that in both fabrication approaches, the incorporation of MOF did not affect the quality of the produced LIG, hence reinforcing the beneficial effect of the doping in terms of the observed electrochemical performance. Continuing with the Raman and XRD analyses results, it was seen that in the case of the LIG-MOF composite, the crystallinity of the MOF was well preserved after the doping of LIG, while in the case of the MOF-LIG composite, the co-lasing process induced a degree of amorphization and disorder on the MOF structure. Finally, the BET surface area analysis showed that the LIG-MOF composite delivered the highest specific surface area of 232.678 m^2^ g^−1^ and the highest pore volume of 0.165 cm^3^ g^−1^ among the three types of electrodes. These results explain why the LIG-MOF composite-based MSC presented the best electrochemical performance.

Next, we establish a comparison of the highest areal capacitance achieved in this work, which corresponds to 4.6 mF cm^−2^ at 0.05 mA cm^−2^ from the LIG-MOF MSC, with other recent works in the field, summarized in Table 2. First, the C_A_ of the LIG-MOF MSC significantly surpasses the value of the LIG MSC of this work (0.29 mF cm^−2^ at 0.05 mA cm^−2^), reiterating the need of LIG doping to enhance its capability in energy storage. Then, to confirm the suitability of the LIG-MOF composite as electrode for supercapacitor applications, we have checked that its C_A_ overcomes, or it is comparable to, the values of other reported LIG-based MSCs using electrodes such as laser-reduced graphene oxide (LrGO) (1.61 mF cm^−2^ at 0.05 mA cm^−2^) [74], Co_1−x_Ni_x_O-LIG (2.17 mF cm^−2^ at 0.7 mA cm^−2^) [75], VO_x_-LIG (2 mF cm^−2^ at 0.25 mA cm^−2^) [76], HfO_2_-LIG (4.5 mF cm^−2^ at 0.04 mA cm^−2^) [77], and LIG-(MOF-199@ZIF-67) (5.0 mF cm^−2^ at 0.2 mA cm^−2^) [78]. We also have added in Table 2 a couple of LIG-based MSCs from the literature where the devices present higher C_A_ values for the sake of fairness: B-LIG MSC (16.5 mF cm^−2^ at 0.05 mA cm^−2^) [79] and lignin-based MoS_2_-decorated LIG MSC (16.2 mF cm^−2^ at 0.1 mA cm^−2^) [80]. Finally, we would like to conclude that having learnt about the potential of Cu_3_(NDI)_3_ MOF as a suitable dopant for laser-induced graphene to enhance its performance as a supercapacitor in this first study, there is room to improve the quality of the LIG-MOF and MOF-LIG composites in future works through further electrode engineering: optimization of the density of deposited MOF on LIG in the case of LIG-MOF material, optimization of the density of deposited MOF on polyimide (Kapton) and of the lasing conditions in the case of MOF-LIG material, and in both processes, trying the doping of the MOF on different laser-induced graphene materials, since the structure of the LIG is fundamental in the final properties of a device.

The relationship between energy density and power density for the LIG, LIG–MOF, and MOF-LIG MSCs is presented in the Ragone plot (Figure 8c). The areal energy and power densities were obtained from Equations (2) and (3). Among the tested devices, the one using LIG–MOF electrodes (red curve) shows the best performance, offering higher energy density than the LIG (black curve) and MOF–LIG (blue curve) supercapacitors, given the similar obtained power density values comparing with the MOF-LIG supercapacitor. The LIG–MOF MSC delivers the highest areal energy density of 60.03 μWh cm^−2^ at a power density of 1292.17 μW cm^−2^. This indicates that integrating MOF on LIG effectively enhances charge storage capacity while maintaining good power output.

Continuing with the Ragone plot (Figure 8c), the devices developed in this work were compared with recent works in the literature. First, comparing the LIG-MOF (red curve) and MOF-LIG (blue curve) MSCs with the devices from references [28] (green point) and [29] (light blue point), where laser-induced graphene was also doped with metal–organic frameworks based materials, it is observed that our LIG-MOF and MOF-LIG supercapacitors show better performance. The reason behind this result lies in the Cu_3_(NDI)_3_ MOF structure. Unlike ZIF-67 [28] and Cu-BTC [29], which employ electrochemically inactive organic linkers, Cu_3_(NDI)_3_ incorporates a redox-active naphthalene diimide (NDI) linker capable of reversible multi-electron redox reactions. This provides an additional pseudocapacitive contribution and enhances charge storage beyond electric double-layer capacitance. Furthermore, the extended π-conjugation of the NDI linker improves electron delocalization and charge transport. Finally, from the Ragone plot, it is observed that the achieved energy and power densities from the LIG-MOF (red curve) and MOF-LIG (blue curve) MSCs are higher than the values obtained from a wide variety of recent LIG-based MSCs: borax-lignin-enriched paper LIG [81], N-doped LIG [82], Fe_2_O_3_ LIG [83], MnO_x_-doped paper LIG [84], Cu-LIG [85], zinc-fluorinated LIG [86], and Ni-Fe-doped carbon cloth-polyethersulfone (PES) LIG [87], which demonstrate that they are competitive candidates for energy storage applications.

## 4. Conclusions

In this study, we successfully synthesized a novel trinuclear copper(I)-based metal–organic framework Cu_3_(NDI)_3_ through a facile solvothermal method using N,N′-bis(3,5-dimethylpyrazol-4-yl)-naphthalene diimide as a structure-directing ligand. Structural and morphological characterizations via XRD, FTIR, SEM, EDX, and BET confirmed the formation of a crystalline porous framework with uniform particle distribution and high surface area, suitable for electrochemical applications.

We further demonstrated the fabrication of good performance supercapacitor electrodes by integrating this Cu-MOF on laser-induced graphene through two different approaches: prior fabrication of LIG and posterior deposition of MOF (LIG-MOF) and prior deposition of MOF on the polyimide precursor and simultaneous irradiation of both elements to form the LIG (MOF-LIG). The results showed that the best device was the LIG–MOF composite supercapacitor, achieving the highest specific capacitance and areal energy density of, respectively, 4.6 mF cm^−2^ at 0.05 mA cm^−2^ and 60.03 μWh cm^−2^ at a power density of 1292.17 μW cm^−2^. This enhancement arises from the synergetic combination of the MOF abundant redox-active sites and the LIG excellent electrical conductivity and hierarchical porosity, enabling efficient ion diffusion and charge transport. The promising results obtained from the electrochemical performance of the LIG supercapacitors doped with the Cu_3_(NDI)_3_ MOF pave the way for developing future works using these materials as potential candidates for efficient energy storage, exhibiting a wide room of improvement to optimize further the doping conditions, test the cycle life of the devices, and try them in advanced flexible applications.

## Figures and Tables

**Table 1:** Summary of the results from the textural analysis of the samples studied in this work.

Sample	SSA (m^2^ g^−1^)	t-Plot Micropore Area (m^2^ g^−1^)	Total Pore Volume of Pores (cm^3^ g^−1^)	Adsorption Average Pore Width (nm)	BJH Adsorption Average Pore Diameter (nm)	BJH Desorption Average Pore Diameter (nm)
MOF	238.346	187.177	0.126	2.112	5.200	5.032
LIG	196.182	125.949	0.164	3.342	11.183	10.953
LIG-MOF	232.678	171.264	0.165	2.847	10.292	10.007
MOF-LIG	138.659	78.439	0.141	4.061	11.814	11.640

**Table 2:** Comparison of the areal capacitance of LIG, LIG-MOF, and MOF-LIG MSCs, fabricated in the present work, with other LIG-based MSCs.

MSC Type	Electrolyte	Current Density (mA cm^−2^)	Areal Capacitance, C_A_ (mF cm^−2^)	Reference
B-LIG	PVA/H_2_SO_4_	0.05	16.5	[79]
MoS_2_-LIG	PVA/H_2_SO_4_	0.10	16.2	[80]
LIG-(MOF-199@ZIF-67)	1M H_2_SO_4_	0.20	5.0	[78]
**LIG-MOF**	1M H_2_SO_4_	0.05	4.6	This work
HfO_2_-LIG	PVA/H_2_SO_4_	0.04	4.5	[77]
**MOF-LIG**	1M H_2_SO_4_	0.05	2.61	This work
VO_x_-LIG	PVA/H_2_SO_4_	0.25	2	[76]
Co_1−x_Ni_x_O-LIG	3M KOH	0.7	2.17	[75]
LrGO	3M NaCl	0.05	1.61	[74]
**LIG**	1M H_2_SO_4_	0.05	0.29	This work

## Data Availability

The original contributions presented in this study are included in the article/Supplementary Materials. Further inquiries can be directed to the corresponding author.

## Supplementary Materials

The following supporting information can be downloaded at https://www.mdpi.com/article/10.3390/nano16030155/s1. Table S1: Crystallographic Data for Cu_3_(NDI)_3_ MOF; Figure S1: (a) N_2_ adsorption/desorption isotherms (BET) and (b) pore size distribution plot of Cu_3_(NDI)_3_; Figure S2: N_2_ adsorption/desorption isotherms of (a) LIG, (b) LIG-MOF and (c) MOF-LIG electrodes. Pore size distribution plot of (d) LIG, (e) LIG-MOF and (f) MOF-LIG electrodes; Figure S3: Cyclic voltammetry at a scan rate of 500 mV s^−1^ of three different samples from (a) LIG, (b) LIG-MOF and (c) MOF-LIG electrodes, to show the good reproducibility of the fabrication processes achieved in this work; Figure S4: Equivalent electrical circuit used for the EIS analysis in this work, consisting in an electric series resistance (R_s_), a charge transfer resistance (R_ct_), a Warburg impedance (R_w_) and the capacitance element (C).
