# Supplementary material for: Innovative Trinuclear Copper(I)-Based Metal–Organic Framework: Synthesis, Characterization, and Application in Laser-Induced Graphene Supercapacitors
Source: Nanomaterials (Basel). 2026 Jan 23;16(3):155. doi: 10.3390/nano16030155 (PMC12899479; doi:10.3390/nano16030155)
Supplement: Supplementary file 1 [file nanomaterials-16-00155-s001.zip › nanomaterials-4103533-supplementary.pdf]

Supplementary Materials

$Cu_3(NDI)_3$	
Chemical formula	$C_{36}H_{24}Cu_3N_9O_6$
Molar mass	869.26
Crystal system	Tetragonal
Space group	I-4
a (Å)	17.33
b (Å)	17.33
c (Å)	33.81
α (°) β (°), γ (°)	90
V (Å³)	10198.7656
Z maille	8
ρ_{calc} (g/cm³)	1.146
l (nm)	1.54060

Table S1. Crystallographic Data for $Cu_3(NDI)_3$ MOF.

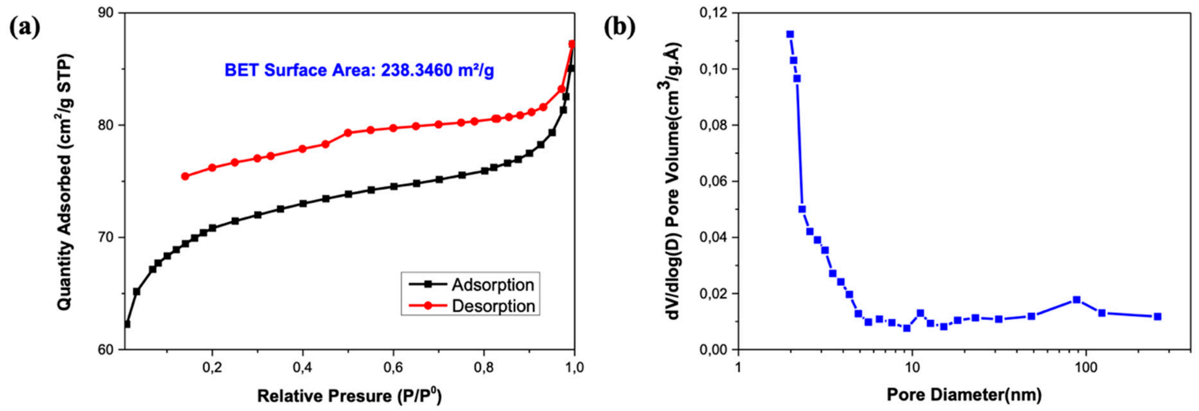

Figure S1. (a) N_2 adsorption desorption isotherms (BET) and (b) pore size distribution plot of $Cu_3(NDI)_3$.

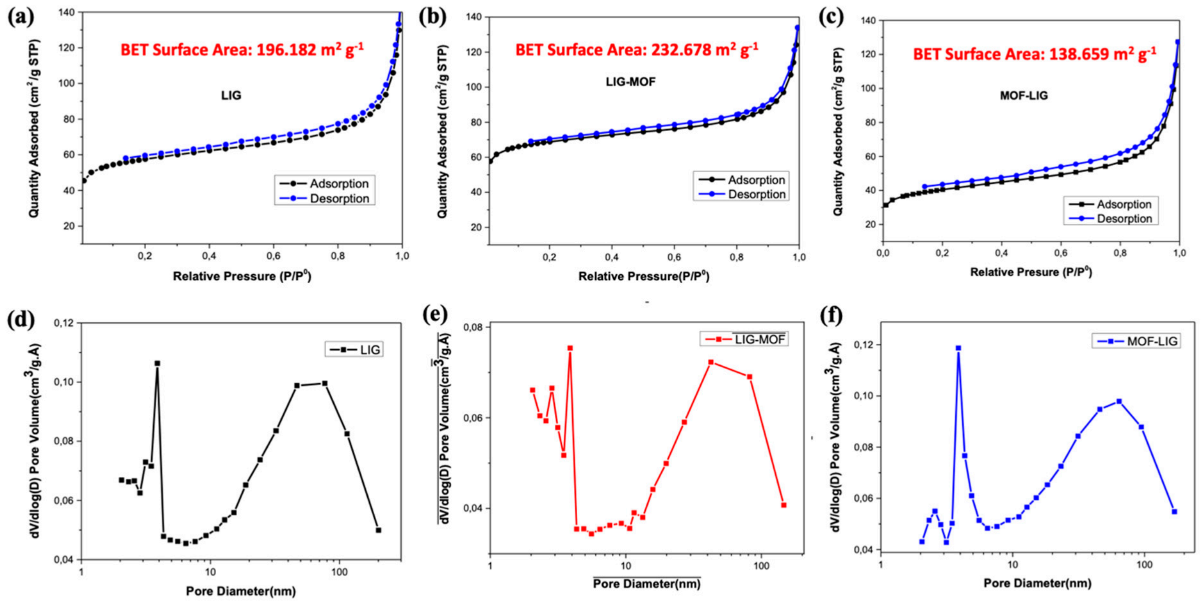

Figure S2. Adsorption/desorption isotherms of (a) LIG, (b) LIG-MOF and (c) MOF-LIG electrodes. Pore size distribution plot of (d) LIG, (e) LIG-MOF and (f) MOF-LIG electrodes.

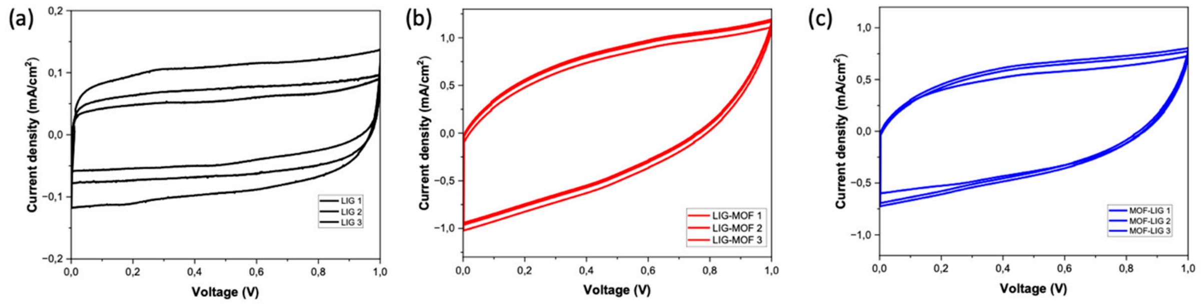

Figure S3. Cyclic voltammetry at a scan rate of 500 mV s⁻¹ of three different samples from (a) LIG, (b) LIG-MOF and (c) MOF-LIG electrodes, to show the good reproducibility of the fabrication processes achieved in this work.

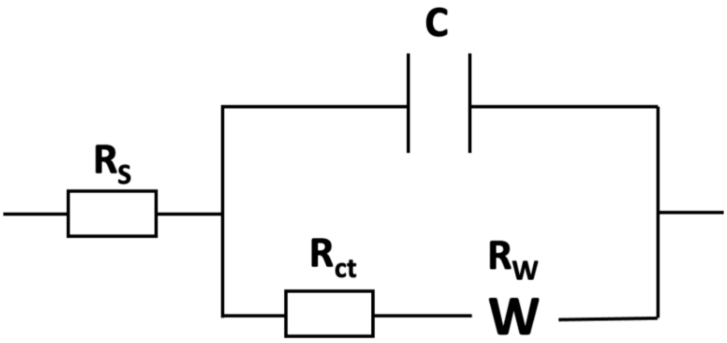

Figure S4. Equivalent electrical circuit used for the EIS analysis in this work, consisting in an electric series resistance (R_s), a charge transfer resistance (R_{ct}), a Warburg impedance (R_w) and the capacitance element (C).
